# A retrospective study on the carbon footprint of bodies donated to science for sustainable medical education and research

**DOI:** 10.3389/fmed.2025.1530121

**Published:** 2025-05-19

**Authors:** Carlo Barausse, Subhi Tayeb, Lorenzo Bonifazi, Simone Lodi, Giulia Adalgisa Mariani, Ester Orsini, Sara Zanni, Alessandra Bonoli, Lucia Manzoli, Stefano Ratti

**Affiliations:** ^1^Centre for Clinical and Surgical Experimental and Molecular Anatomy, and Cellular Signaling Laboratory, Department of Biomedical and Neuromotor Sciences, University of Bologna, Bologna, Italy; ^2^Unit of Oral Surgery, Department of Biomedical and Neuromotor Sciences, University of Bologna, Bologna, Italy; ^3^Department of Management, University of Bologna, Bologna, Italy; ^4^Department of Civil, Chemical, Environmental and Materials Engineering, University of Bologna, Bologna, Italy

**Keywords:** human anatomy, body donation, carbon footprint, medical education, medical research

## Abstract

**Introduction:**

A comprehensive understanding of human anatomy is essential for medical education, with donated human body dissection remaining the gold standard for this purpose. However, in countries where there is a shortage of locally donated bodies, anatomical centers are increasingly turning to external body donation programs, such as those in the United States, to meet their needs.

**Methods:**

This study assesses the carbon footprint (CO2e) of locally sourced vs. internationally sourced donated bodies, with the carbon footprint of the latter being estimated hypothetically. A retrospective observational study using Life Cycle Assessment (LCA) methodology was conducted to evaluate the environmental impacts. The analysis included factors such as transportation, preservation methods (fresh vs. embalmed), and additional aspects such as refrigeration and aeration.

**Results:**

Locally donated bodies had an average transport distance of 201.19 ± 172.78 km, resulting in 14 ± 11.84 kgCO2e per body. In contrast, international transport from the US hypothetically produced approximately 450.375 kgCO2e per body, representing a 3114.3% increase. The total carbon footprint for a locally donated body was 8948.99 kgCO2e annually. These findings suggest that local donation programs could significantly reduce transportation emissions, making them more eco-friendly.

**Discussion:**

Promoting local donation programs could not only enhance educational opportunities but also minimize the environmental impact of anatomical studies. Increasing the number of local donors would optimize the use of management systems, such as aeration and refrigeration, further improving sustainability. Due to the limitations of this study, further research is needed to refine these findings and explore strategies for reducing the carbon footprint in medical training.

## 1 Introduction

A deep understanding of human anatomy is essential for medical professionals and serves as a critical milestone in both pre-graduate and post-graduate programs, including during the study and training phases and for medical research purposes (1). One effective way to learn and practice is through donated human body dissection, which remains an excellent method to achieve a high-fidelity and realistic three-dimensional comprehension of human anatomy (2, 3). Recent studies have also highlighted the importance of improving legal and ethical frameworks surrounding body donation, as these regulations vary significantly across European countries, impacting the availability of donated bodies for medical education and research (4).

Nonetheless, in Italy, a shortage of donated bodies has been observed compared to the growing number of medical students (5–7). To facilitate anatomy studies and address the challenges of limited donated body availability, various types of artificial models or other technological aids have been explored as substitutes in recent years. Many universities are currently using synthetic models for their students due to their ease of management and availability. Artificial models can, therefore, be useful in supplementing anatomical studies on donated human bodies, especially at the undergraduate level, where topics are approached with less complexity (8). Each synthetic model can usually provide a specific type of simulating procedure; for instance, there are models designed for basic life support, as well as others tailored to various anatomical systems such as the cardiovascular, musculoskeletal, and nervous systems. These models offer valuable hands-on experiences, aiding students in developing a comprehensive understanding of different aspects of human anatomy. However, despite the continuous technological advancements in synthetic models, traditional human body dissection remains unmatched in terms of educational effectiveness (9). Indeed, while the use of artificial models can still be effective and beneficial, especially in undergraduate courses where exercises are generally less complex, the use of donated human body dissection often becomes inevitable for postgraduate training (10). In undergraduate settings, anatomy based on donated human bodies allows students to develop not only a solid understanding of human structure but also essential professional and ethical values, such as respect for the human body, empathy, and awareness of death as part of clinical reality. These humanistic aspects are a fundamental part of the educational impact of body donation, which goes far beyond the purely technical learning dimension. While institutional models may vary, this integrated approach aligns with a growing recognition of the ethical and pedagogical relevance of human dissection in shaping responsible future physicians.

Nevertheless, once the different possible applications of various options are established, there is no doubt that the study, training, and research using donated-to-science human bodies remain the gold standard in all medical fields (11).

A potential alternative solution to address the shortage of donated human bodies to science at certain anatomical centers could involve adopting external donation programs, where donated human bodies or specific anatomical parts are sourced from foreign countries (e.g., the United States) (12). In this context, another relevant aspect to consider is the ecological sustainability of the dissection process and how it might be made more environmentally responsible. Given the growing concerns surrounding global climate change, there is increasing awareness of the environmental consequences of human activities. This has led to a broader recognition of the need to reflect on the ecological impact of various choices, including the materials we use and the decisions we make (13). As sustainability becomes an increasingly important consideration, it seems worthwhile to explore how the dissection of donated human bodies could be approached in a more environmentally friendly manner.

One area that warrants further exploration is the environmental impact associated with the life cycle of donated human bodies, including transportation, preservation, and the dissection itself. These factors can contribute to the overall carbon footprint of anatomical education. While the scientific value of body donation is clear, it would be useful to investigate whether there are opportunities to reduce its ecological footprint.

Moreover, these considerations align with the broader discourse surrounding the environmental footprint of medical education and could help foster a more inclusive discussion about the future of anatomical studies. Such a dialogue might encourage the development of practices that integrate sustainability without compromising the quality of education. Drawing inspiration from the well-known Latin expression, “Hic mors gaudet succurrere vitae,” an updated version that reflects contemporary concerns may be proposed: “Hic mors gaudet succurrere vitae et planetae.” This revised phrase could symbolize not only the intersection of the human life cycle and education but also the potential importance of environmental responsibility in contemporary anatomical practices, an aspect that will be investigated in this study.

This study aimed to evaluate the carbon dioxide equivalent (CO₂e) emissions linked to the use of donated human bodies from a local Italian donation program, compared to a hypothetical scenario where the same bodies would be obtained through an international donation program.

## 2 Materials and methods

All bodies included in the analysis, sourced from the local program, were donated to the Centre for Clinical and Surgical Experimental and Molecular Anatomy, Department of Biomedical and Neuromotor Sciences, University of Bologna, one of the Italian reference center for the conservation and use of bodies donated post-mortem for study, training, and scientific research purposes. The United States body donation program was used for comparison. The reference to the United States was chosen due to its frequent use as an external source of human anatomical bodies/parts in countries facing donor shortages, such as Italy. The international transport scenario described in this study was constructed as a hypothetical model, relying on general estimations of distances and associated emission.

### 2.1 Study design and data collection

A retrospective observational study was conducted in a blinded manner. To evaluate the carbon footprint of a donated human body, the journey of the donated body was traced, starting from the geographical origin of the donor and including the fresh or embalmed preservation methods. The first technique involves preservation relying on refrigeration only (−20°C) to slow down decomposition, while embalmed donated bodies undergo a traditional preservation process using chemicals, typically formaldehyde, to fix tissues and prevent decay.

Data were anonymously collected, including information on local (Italy) geographical origin, means of transport, distance traveled (in kilometers), preservation method (fresh or embalmed), number of refrigeration days at −20°C and +4°C, final geographical destination, mode of transport, and the carbon footprint for cremation.

The analysis focused on key components of the process, including embalming, cold preservation, transportation, the use of anatomical consumables, wastewater management, and the operation of aeration systems. Emissions were reported in terms of carbon dioxide equivalents (kgCO₂e) using the IPCC GWP100 method over a 100-year time horizon. Emission factors were primarily derived from the Ecoinvent 3.8 database (APOS, Unit Process system model), and electricity emissions were calculated according to the Italian country-specific energy mix.

The embalming procedure involved 10 liters of fluid per body, composed of formaldehyde (1.83%), phenol (27.8%), ethanol (33%), and glycerol (33%). The amount of each component was calculated based on its respective density and converted into mass. The environmental impact of these substances was estimated through the corresponding Ecoinvent entries, considering both their production and disposal through hazardous waste incineration with energy recovery.

Cold preservation was modeled independently of embalming. Refrigeration chambers were assumed to have a useful life of 10 years, and the daily electricity consumption per body was determined based on institutional data. Two types of chambers were considered: freezers operating at −20°C and refrigerators at +4°C. Their respective average daily electricity consumption was 11.232 kWh and 12.6 kWh per body, which, when combined with the Italian electricity emission factor (0.39896 kgCO₂e/kWh), resulted in a marginal daily impact of approximately 0.000064 kgCO₂e per body.

Transportation of donated bodies to the anatomical center in Bologna was carried out exclusively via refrigerated trucks. An average body weight of 75 kg was assumed. An emission factor of 0.000464 kgCO₂e per kilogram per kilometer was calculated. In the hypothetical international model, based on a United States donor program, an additional 500 km of refrigerated truck transport was included, followed by an intercontinental flight from Chicago to Bologna covering 7,500 km. The flight emissions were estimated using a standard cargo emission factor of 0.602 kgCO₂e per kilogram per kilometer.

The use of anatomical consumables such as nitrile gloves, nylon-based materials, PET containers, and polyethylene packaging film was also included in the model based on actual consumption records from the anatomy center. Wastewater from anatomical cleaning and preparation procedures was calculated based on Ecoinvent’s values for average wastewater treatment in Europe.

The energy consumption of ventilation and air treatment systems used in the dissection rooms was included under a worst-case scenario assumption. Annual electricity consumption from HVAC and aeration systems was estimated at 709,632 kWh applying the Italian emission factor for medium-voltage electricity.

### 2.2 Life cycle assessment (LCA)

This study developed a streamlined Life Cycle Assessment (LCA) to compare different options for the sourcing of donated-to-science bodies for educational and research purposes, specifically comparing local and international sourcing. The LCA methodology applied is compliant with international standards (ISO 14040) and was developed using SimaPro software. The LCA framework encompasses all stages of the life cycle. Key environmental indicators such as greenhouse gas emissions, energy consumption, and waste generation are quantified for each scenario, but a special focus was posed on carbon emissions, expressed in terms of carbon dioxide equivalent (CO2e). For this reason, the calculation method applied is IPCC100, widely adopted for quantifying greenhouse gas emissions and their impact over a 100-year time horizon. Developed by the Intergovernmental Panel on Climate Change (IPCC), this method assigns global warming potential (GWP) values to various greenhouse gases based on their radiative forcing and atmospheric persistence over a century. The IPCC100 is particularly valued for its standardized methodology, allowing for comparability across studies and industries. By focusing on a 100-year impact window, the method provides a balanced perspective on both immediate and long-term climate effects, facilitating informed decision-making in sustainability and policy development. Data collection integrated primary data, directly collected from the program manager, and secondary data. The reference database for the sourcing of non-primary data is Ecoinvent v.8.1.

### 2.3 Bioethical study approval

The present study received approval from the University of Bologna School of Medicine bioethical board (Prot. N. 0102300 of 10/04/24). The bodies donated to science included in this study are part of the Italian National Program for Body Donation to Science (Law N. 10 of 10 February 2020). The donors provided consent for research and training activities. The body donors were treated with the utmost respect and in accordance with the recent guidelines issued by the International Federation of Associations of Anatomists. The study was conducted in agreement with the EU-GDPR and the Helsinki Declaration. All data were collected anonymously.

### 2.4 Statistical analysis

The 1-year carbon footprint of a single donated body was used as the statistical unit for analysis. A descriptive statistical analysis was performed to summarize the data, including categorical variables presented as absolute and relative frequencies and continuous variables reported as mean, standard deviation, range, and median. The normal distribution of the variables was assessed using the Kolmogorov–Smirnov test, while homoscedasticity was evaluated using Levene’s test. Data were analyzed using Statistical Software Stata 18 (StataCorp. 2023, Release 18. College Station, TX: StataCorp LLC).

## 3 Results

A total of 32 donated bodies were included in the study.

**Figure 1:**
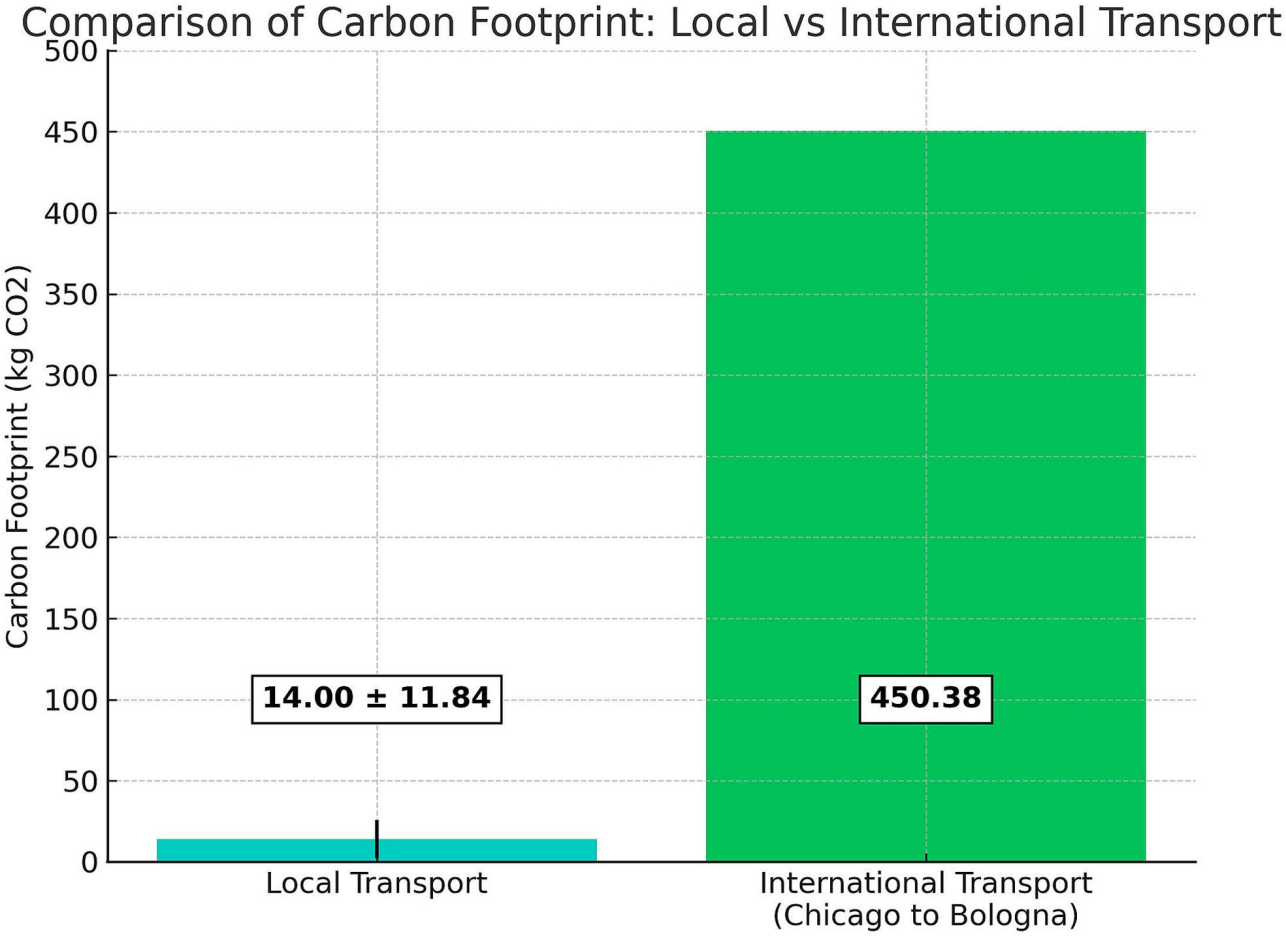
Bar chart illustrates the kgCO2e comparison between the emissions of donated bodies transported locally to the Anatomical Centre in Bologna and those transported internationally from the United States. The international transport (Chicago to Bologna) is estimated at 450.38 kgCO2e, while the local transport has a mean value of 14.00 ± 12.03 kgCO2e. The error bar represents the standard deviation for local transport.

In the transportation phase to the Anatomical Centre in Bologna, all donated bodies were ground-transported using refrigerated trucks over an average of 201.19 ± 172.78 km, resulting in a mean CO2 production of 7 ± 6.01 kg (kgCO_2_e). The same values were considered for the return journey to their geographical origin, leading to a total average CO2 production of 14 ± 11.84 kgCO2e per body for transportation (Figure 1).

The carbon footprint of the embalming process resulted in total emissions of 22.73 ± 20.36 kgCO₂e per body. This value is derived from the sum of individual contributions: 0.199 kgCO₂e from formaldehyde (1.83%), 2.98 kgCO₂e from phenol (27.8%), 2.59 kgCO₂e from ethanol (33%), and 4.16 kgCO₂e from glycerol (33%). Additionally, the incineration of unused embalming fluid, modeled as hazardous waste with energy recovery, was included in this estimate.

Refrigeration days at −20°C averaged 112.03 ± 139.62 in 1 year, while at + 4°C, the mean was 132.91 ± 120.61. The energy consumption of refrigeration chambers contributed minimally to the total footprint. Based on measured electricity use per day per body (11.232 kWh for −20°C and 12.6 kWh for +4°C), and applying the Italian electricity emission factor (0.39896 kgCO₂e/kWh), the mean carbon footprint for refrigeration over 100 days was estimated at 0.016 ± 0.004 kgCO2e kgCO₂e per body per year.

**Figure 2:**
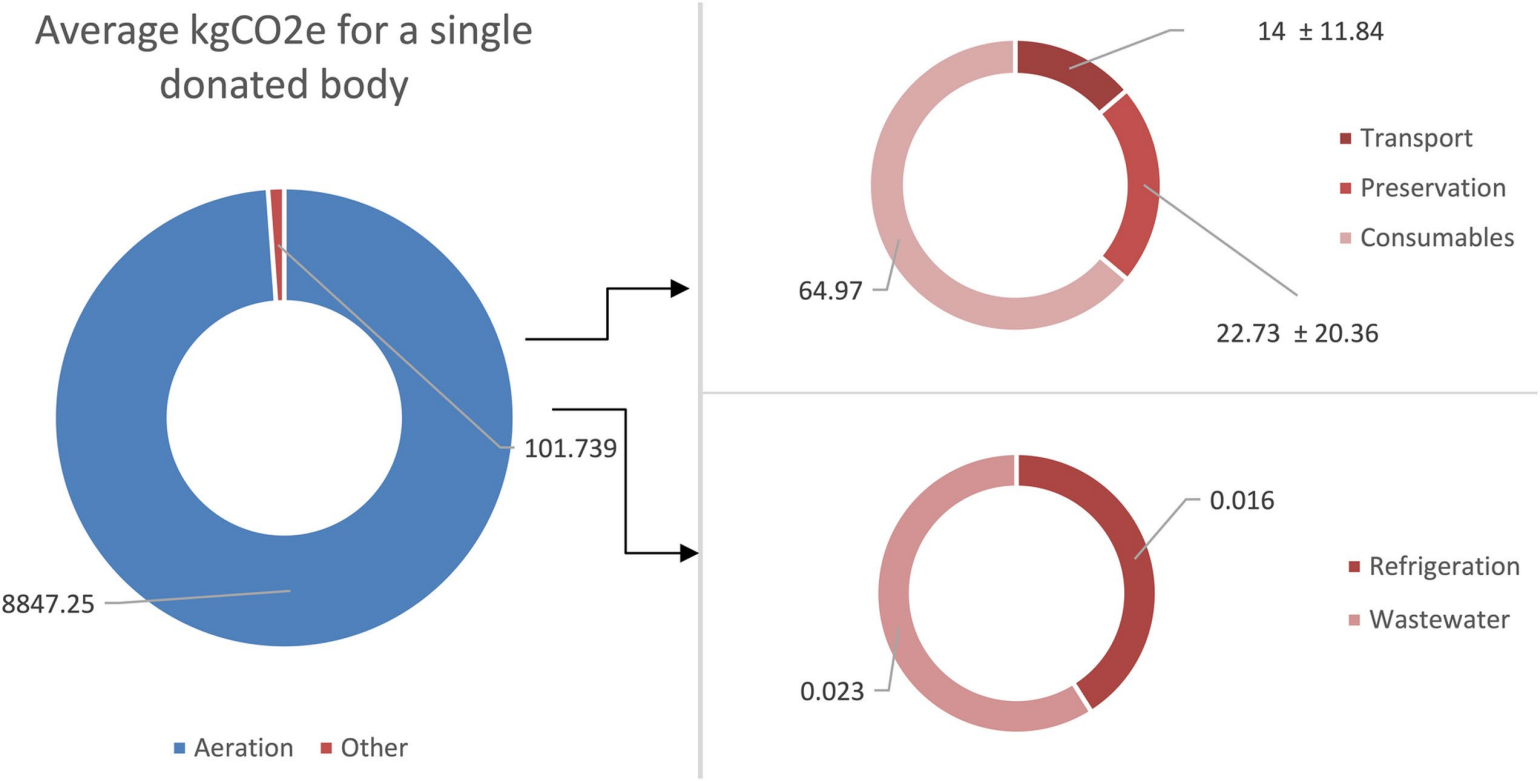
Pie chart shows the kgCO2e per single body donated to science, divided into various activities including aeration, transport, preservation, consumables, refrigeration, and wastewater.

Considering these factors, the cumulative carbon footprint for a single donated body was calculated at 36.74 ± 22.03 kgCO2e (Figure 2).

In addition, other factors related to educational activities contributed to the overall carbon footprint, including consumables, wastewater treatment, and aeration. The consumables related to a single donated body produced an estimated 64.97 kgCO2e. This estimate assumed the production and final disposal (incineration as hazardous waste) of a mix of plastic materials. Specifically, wastewater treatment produced an estimated 0.023 kgCO2e per body, based on a standard wastewater treatment process. The operation of the HVAC and aeration systems represented the single largest source of emissions. The total annual electricity consumption of 709,632 kWh (including inverter and refrigeration units) translated into 283,112 kgCO₂e per year using the Italian energy mix. When divided by the number of bodies handled annually, the estimated footprint was 8,847.25 kgCO₂e per body (Figure 2).

Taking all these factors into account, the total estimated carbon footprint for a single human body’s activity over 1 year amounted to 8948.99 kgCO_2_e.

This footprint was hypothetically compared to an estimate for an identical number of donated bodies, assuming they were sourced from the United States human body donation program. The international transportation from Chicago to Bologna, by aircraft over 7,500 km and refrigerated lorry for 500 km in the US, results in approximately 6.005 kgCO_2_e per kg of human body transported. The analysis modeled the international shipment of a human body as air cargo, applying standard emission factors from the Ecoinvent 3.8 database, consistent with the Life Cycle Assessment methodology. These factors allocate emissions per transported kilogram of cargo, without including passenger-related overhead. Given an average human body weight of 75 kg, this translates to approximately 450.375 kgCO_2_e for transportation alone for a single human body. International transport from the US produced approximately 450.375 kgCO_2_e per body, representing a 3114.3% increase compared to the local program (Figure 1).

Moreover, donated bodies transported from the US are often used as specific body parts rather than whole bodies, potentially further increasing energy consumption and CO2 output. These segments are transported to different locations based on the specific needs of the courses, thereby theoretically increasing the cumulative carbon footprint.

## 4 Discussion

Within the field of medicine, a profound understanding of human anatomy remains not only imperative in pre-lauream education but also across every specialization (14). From ancient times, the exploration of the human body and its physiology has involved the practice of human body dissection. Despite being an ancient science, it continues to hold pivotal importance in the present day (4).

Indeed, the donated human body still represents the gold standard in the study of anatomy today because of its similarity to a living being (11). Moreover, with new technologies, such as the full-body revascularized and ventilated specimen, the donated body can currently be considered suitable for simulating surgeries with high specialist and realistic value (15). Especially in research and the development of new surgical methods and techniques, the donated-to-science human body can be likened to the “first patient,” thereby reducing potential risks or complications in living subjects (16). For the practice of intricate surgical procedures, the importance of human body dissections is unquestionable. While synthetic models may be hypothetically useful in very preliminary anatomical studies, they are not suitable for research on new surgical techniques or the development of new medical devices. Similarly, for students or recent graduates, the human body can represent the perfect “first patient” for surgery, providing both the safest and most reliable simulation (17).

Currently, many universities, including those in Italy, are facing a systematic shortage of donated bodies and reserve their use for moments of indispensable importance (18). Regarding the exploitation of animal models, they are not comparable in terms of efficacy, in addition to the ethical considerations that need to be raised due to the sacrifice of animals. Artificial models are mainly used in broad courses with numerous students, where having a donated human body for each trainee would not be feasible. In this first-phase learning, the synthetic model could serve as a viable option to bring the student closer to the donated human body and be preparatory to a more complex and realistic anatomy, being a halfway between the bi-dimensionality of books and the dissection (9). However, their use is often limited to a narrow and simplified view of anatomy, making them more appropriate for undergraduate courses rather than for actual surgical simulations. Moreover, these models are mostly designed for specific types of learning or particular techniques, such as the Basic Life Support (BLS) training, resulting not as an alternative to donated human bodies but as being complementary (8).

Having assessed that the donated-to-science human body remains the gold standard for educational purposes, in cases of local donor shortages, a possible solution for some anatomic centers could be to apply for an external body donation program. The United States is currently one of the more common origin places for donated bodies. The long journey adds significantly to the carbon footprint; for example, a Chicago to Bologna flight may add 450.375 kgCO_2_e per donated body, resulting in more than 3,116,96% of the total carbon footprint of a single human body. A lower but still substantial percentage may be attributed to long-distance national transportation, which is what commonly happens in a national reference center such as the Centre for Clinical and Surgical Experimental and Molecular Anatomy of Bologna (14). Assuming that emissions related to all imports coming from the United States in 2009 were 210.3 Mt. CO2, we estimated that donated bodies would account for 0.000007% of the total. In contrast, the local donation program represents 0.0000006%. Although this may be a small fraction of the total, there is a difference of 0.0000064%, with the emissions from U.S. imports being approximately 11.67 times greater than the local donation program.

To minimize the carbon footprint, the principles of environmental sustainability—known as the 3 R’s: reduce, reuse, and recycle—could be applied. By reducing the need for long-distance transportation (reduce), maximizing the utilization of each donated body through multiple educational, training, and research sessions (reuse), and giving a body already existing in nature a new life and purpose by donating it to science (recycle), sustainable practices within this context could be promoted. As a result, the carbon footprint is primarily associated with the management and transportation of the donated body. For this reason, local donation programs should be prioritized as much as possible to reduce transportation emissions. Moreover, since management is not strictly related to the number of bodies—such as the aeration and refrigeration systems, which are integral to anatomy rooms— having more donors would reduce the carbon footprint per body and provide more students and trainees the opportunity to learn in an extremely realistic setting.

**Figure 3:**
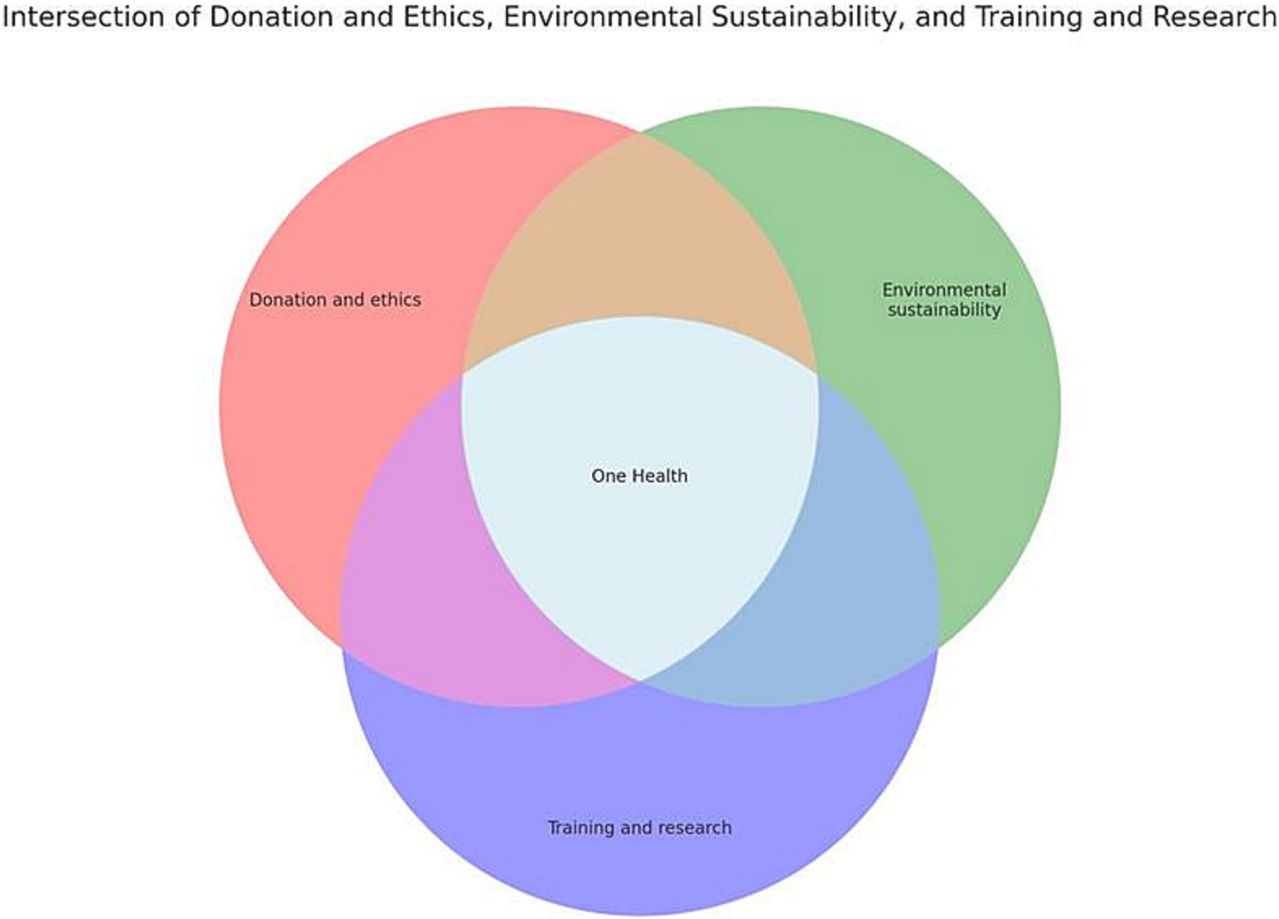
Venn diagram of the one health concept applied to the local human body donation program.

Adopting the One Health approach could further address these challenges. One Health is a collaborative, multisectoral, and transdisciplinary strategy that recognizes the interconnection between human health, animal health, and environmental health. In the context of anatomical education, this approach emphasizes integrating training and research with ethical donation practices, ensuring health and safety standards, and promoting environmental sustainability. By fostering local body donation programs, we not only enhance educational and research opportunities but also reduce environmental impacts, aligning with ethical considerations and safety protocols. This holistic perspective supports the advancement of medical science while safeguarding ecological balance and public health (Figure 3).

To promote body donation and raise awareness of the expanding body donation program in Italy and beyond, particularly within the general public, various strategies could be implemented, including the co-creative use of graphic medicine and novels. (19, 20).

Nonetheless, while the U.S. body donation program primarily involves single body parts to meet specific training requests, considering the donation of whole bodies could further reduce the carbon footprint and enhance the re-use of each body (21). It is important to note that the return of remains to families, which is common in many U.S. academic donor programs, was not included in our model due to the lack of consistent data. This omission likely results in an underestimation of the true environmental impact of internationally sourced donations.

A recent study provides valuable insights into the geographical impact of human gift registries, particularly in the context of centralized resources for anatomy education. The article offers a detailed model for understanding how the geographical distribution of body donations influences the logistics of human anatomy programs. By examining the distances traveled by body donations, the study highlights the potential for localized donation systems to reduce transportation-related environmental impacts, further confirming the results of the present paper. The findings are highly relevant to our study as they underscore the importance of considering geographical factors when assessing the sustainability of body donation programs (22).

This study introduces a preliminary ecological analysis of the body donation process. This topic, if further explored, could potentially be discussed in best practices consensus documents for the dissection room quality system and in human body donation programs’ best practices and recommended standards (23, 24).

Recent discussions on sustainability in medical education have highlighted the potential benefits, according to the present study, of localizing anatomical body donation programs. A recent study emphasizes the importance of considering environmental sustainability within anatomy education, suggesting that reducing the carbon footprint of anatomical studies could be achieved through more localized approaches (25). Furthermore, while the international transport of human bodies could raise ethical concerns, some authors point out that these practices might require further ethical considerations and the development of guidelines to ensure respectful management (26). In this context, our study may support the notion that promoting local body donation programs could not only reduce environmental impact but also help address these ethical considerations by minimizing the need for international transportation.

However, it is crucial to consider the limitations of this study, given its retrospective nature and the fact that the donated human bodies involved are related to a single specific Italian center. In particular, the study relies on data from a single Higher Education Institution Dissection Room. The focus of the study was on this specific context, but the importance of incorporating broader data at a national or EU level in future research would be valuable. Expanding the scope of data would provide a more comprehensive understanding of body donation practices across different regions. Moreover, due to the complexity of the whole process, some parameters were estimated by considering the worst-case scenario (e.g., aeration), which may not be fully representative of everyday practice.

A key limitation of this study is the lack of primary data on donated bodies sourced from the United States Body Donation Program, with data being estimated hypothetically to construct a generalized model. Even within this context, the comparison with the U.S. body donation process can still offer valuable insights, particularly in terms of screening LCA. This remains an important aspect to consider when interpreting the findings. Nevertheless, this study could lay the foundation for future research and introduce an important concept that may stimulate further discussion and studies on environmental awareness.

## 5 Conclusion

The donated human body remains the gold standard for educational and training purposes in medicine, as it already exists in nature. Since the majority of the CO2 emissions are related to the management and transportation of the donated bodies, a national and local awareness-raising campaign should be supported to help reduce its carbon footprint. The phrase “Hic mors gaudet succurrere vitae et planetae” (This is the place where death delights in helping the living and planet) could be even truer with a greater number of donated bodies sourced from local areas. To conclude, human body donation should be considered not only a philanthropic choice but also an eco-friendly one.

## Data Availability

The raw data supporting the findings of this study will be made available from the authors upon reasonable request.
